# On the Irritable Uterus

**Published:** 1829-10-01

**Authors:** 


### On the Irritable Uterus. By *Robert Gooch*, M.D. &c.

[Art. IV.]

The fifth chapter of Dr. Gooch's work is occupied with the subject of irritable uterus—a painful and tender state of this organ, neither attended by, nor tending to produce, change in its structure. Dr. G. at first took the disease for chronic inflammation, which might end in disorganization; but further observation, though it taught him that the complaint was very intractable, proved that it was not a disorganizing malady. He is acquainted with cases where it has now lasted more than ten years, and yet where the uterus is unaltered in structure.

"Pain in the lowest part of the abdomen and loins attends various diseases of the unimpregnated uterus. It is the chief symptom in painful menstruation; but here it occurs only during the menstrual period, and is quite absent during the rest of the month. It is the most distressing symptom in descent of the uterus, (prolapsus,) but here it occurs only in the upright posture and exercise, ceases on lying down, and replacing the organ, and is prevented by supporting it in its natural situation. It attends most of the diseases of structure to which the uterus is liable; but the change of structure, which may be ascertained by examination, distinguishes the nature of the pain.

"A patient who is suffering from the irritable uterus complains of pain in the lowest part of the abdomen, along the brim of the pelvis, and often also in the loins. The pain is worse when she is up and taking exercise, and less when she is at rest in the horizontal posture; in this respect it resembles that of prolapsus uteri, but there is this difference, that in the latter, if the patient lies down, she soon becomes quite easy, but in the complaint of which I am speaking, the recumbent posture, although it diminishes, does not remove the pain. It is always present in some degree, and severe paroxysms often occur, although the patient has been recumbent for a long time. If the uterus is examined, it is found to be exquisitely tender, the finger can be introduced into the vagina, and pressed against its sides without causing uneasiness, but as soon as it reaches and is pressed against the uterus, it gives exquisite pain. This tenderness, however, varies at different times, according to the degree of pain which has been latterly experienced. The neck and body of the uterus feel slightly swollen, but this condition also exists in different degrees, sometimes sufficiently manifest, sometimes scarcely or not at all perceptible. Excepting, however, this tenderness, and occasionally this swelling, or rather tension, the uterus feels perfectly natural in structure; there is no evidence of scirrhus in the neck, the orifice is not mis-shapen, its edges are not indurated. The patient, finding her pain greatly increased by rising and walking, soon learns to relieve herself by lying on the sofa, and at length spends nearly her whole time there. Notwithstanding this precaution, there is always a considerable degree of uneasiness, but this frequently increases to severe pain. These paroxysms generally come on either a few days before menstruation, or (as is the case in many instances) a few days afterwards. If the paroxysm is properly treated, it subsides in a few days to the ordinary and more moderate uneasiness. Whilst this uneasiness is felt in the substance of the uterus, the general circulation is but little disturbed. The pulse is soft, and not much quicker than is natural; but it is easily quickened by the slightest emotion. In a few instances, however, there has been a greater and more permanent excitement of the general circulation; the degree in which the health has been reduced has been different in different cases. A patient who was originally delicate, who has suffered long, and has used much depleting treatment, has been (as might reasonably be expected) the most reduced; she has grown thin, pale, weak, and nervous; menstruation often continues regular, but sometimes diminishes, or ceases altogether; the functions of the stomach and bowels are not more interrupted than might be expected from the loss of air and exercise; the appetite is not good, and the bowels require aperients; yet nothing more surely occasions a paroxysm of pain than an active purgative. Such are the leading symptoms of this distressing complaint. To embody them in one view, let the reader fancy to himself a young or middle aged woman, somewhat reduced in flesh and health, almost living on her sofa for months, or even years, from a constant pain in the uterus, which renders her unable to sit up and take exercise; the uterus, on examination, unchanged in structure, but exquisitely tender; even in the recumbent posture always in pain, but subject to great aggravations more or less frequently. 314.

The causes are supposed to be, considerable bodily exertions at times when the uterus is in a susceptible state. In one patient it came on after an enormous walk during menstruation—in another by going a shooting, with her husband, soon after an abortion—in a third, by standing many hours, for several nights in succession, at concerts and parties—in a fourth, by a long journey over the paved roads in France, and so on. But the above appeared to be the *exciting* causes, there were doubtless *predisposing* ones in operation previously.

Long-continued pain in an organ liable to malignant diseases leads, of course, to apprehension of organic changes, and to repeated examinations that end in no discovery, except that of tenderness or some slight swelling. The recovery is often extremely tedious—and relapses are very common.

"By complete repose in the recumbent posture, and proper remedies, the painful paroxysms become slighter, and return at longer intervals; the stationary uneasiness becomes gradually less, and at length ceases altogether, and at the end of a few months the patient is left free from pain, but more or less enfeebled. No disease, however, is so liable to relapse. The patient, feeling easy, finding herself feeble, and supposing that air and exercise are necessary to the recovery of her health, rises and goes about again, and after a short interval of caution, throws aside her fears, engages in walks, drives, and gaiety, or takes a journey to the sea, for the recovery of her health. This conduct commonly occasions a complete relapse, and the patient and her attendant are again involved in their former suffering, apprehensions, and difficulties." 316.

What is the nature of the disease? Mr. Brodie has described an irritable or hysterical knee—Sir Astley Cooper an irritable mamma—and we believe that the same term is applicable to the uterus—and the same explanation to be given of its nature.

The treatment which our author has found most useful (tardy as it is in some cases, and ineffectual in others) consists, in subduing pain and restoring the general health. The difficulty is to know when to discontinue the former indication, and when to aim at the latter. The remedies for subduing pain are, the horizontal posture, narcotics, warm hip-baths, occasional local bleeding, to which may sometimes be added mercury and counter-irritants.

"In all those cases in which the pain is perpetual, repose should be perpetual. The patient must abstain not only from foot and carriage exercise, but from the upright posture, which even for short intervals is often sufficient to counteract the cure. As soon as she is dressed in the morning, she should be placed on her sofa with her shoulders as low as the pelvis, and in that posture remain the whole day. At first it is tedious, but she soon learns to amuse and occupy herself in this position, to write, read, work, and draw. This posture more or less strictly observed (the degree of strictness being soon taught by the sensations of the patient) is absolutely necessary for the completion of the cure, not only till all pain has ceased, but for some time afterwards, and even then must be relinquished with the utmost caution or rather timidity." 319.

The next measure is to draw blood. As the general circulation is seldom disturbed, local is preferable to general bleeding. Cupping affords more relief than leeching. The best part to apply the glasses is the upper part of the sacrum—or the part to which the pain is referred. Leeches afford most relief when applied to the hæmorrhoidal vessels, or between the labia pudendi. The quantity drawn must depend on circumstances—the best guides being the state of the constitution, the duration of the disease, and the relief afforded.

"Twelve ounces of blood is a large local blood-letting; it seldom requires to be repeated to the same amount; I have known four ounces afford all the relief which this remedy was capable of effecting. But the pain, if only diminished will increase, and if removed, will return sooner or later. The blood-letting, therefore, requires to be repeated, and the question is, whether to defer it until the return or increase of the pain, or to anticipate this return or increase. I think the latter is preferable when the period of recurrence can be nearly calculated; both reason and experience show that a mode of treatment which prevents an organ from resuming its morbid action, is more likely to remove it permanently, than one which permits the action to recur, and then removes it; the subsequent bleedings, however, ought not to be so large as the first, and as it may be necessary to repeat these several times, it is important to discover the minimum of blood which will afford relief." 321.

"The next remedy is narcotics. Of such medicines the most useful are, one-third camphor and two-thirds extract of henbane, or hemlock, or poppy, divided into pills of five grains; of which one may be taken two or three times a day :—or, about ten grains of extract of poppy, dissolved in an ounce of gruel, may be injected into the rectum every day, immediately after the bowels have acted."

As want of exercise and narcotics confine the bowels, aperients must be used; but they should be of the gentlest kind. "That is the best aperient which will act only once plentifully, and without pain; and those which have most frequently acted in this way have been the solution of sulphate of magnesia in infusion of roses—castor oil—electuary of senna—sulphur." One of these should be taken every second day. The horizontal posture and local bleedings, with narcotics, are the remedies most invariably useful. There are other auxiliaries, however, which are not to be neglected. One is the warm hip-bath, at a temperature of 96°, for half an hour every, or every other night. But a better still is the partial steam-bath. The flannel sack should be drawn up to the præcordia, so as to enclose the abdomen and lower extremities, and these may be exposed to the action of the steam for half an hour every other day.

"Another remedy is a mild course of mercury; from three to five grains of blue pill, or compound calomel pill, mixed with five grains of extract of henbane, taken every night for several weeks, or every other night for many weeks, have sometimes, without affecting the gums, occasioned a very regular action of the bowels; and during its influence, the periodical aggravations of pain have not recurred, and the permanent pain has diminished, and at length ceased altogether. Whilst the mercury has had this favourable influence over the local disease, it has occasioned no material injury to the constitution. This has been its effect chiefly when the health has not been much reduced, and the disease has not lasted very long. On the contrary, in other cases, in which the body has been previously debilitated and emaciated, mercury, although it had a favourable influence over the local disease, occasioned so much wasting, weakness, and nervousness, as to compel me to discontinue it. Mercury requires to be employed with the utmost circumspection, and its effects in each individual case ought to determine whether it should be persisted in or discontinued." 324.

Counter-irritation is another remedy which deserves notice. Small blisters, the size of a watch, may be applied to the upper part of the sacrum—allowed to heal—and then re-applied many times. Or a caustic issue, the size of a dollar, and kept open with savin ointment, may be established on the same part. In very sensitive constitutions, these blisters excite much irritation, without equivalent good.

"The practitioner can do no harm, and must do good as long as he confines himself to the employment of the recumbent posture, mild narcotics, warm hip-baths, and unirritating aperients; but with blood-letting and mercury he must be more cautious. I have seen many cases in which the pain was rather aggravated than relieved by bleeding; the patient rendered weak and irritable, and the effect of the remedy was unmixed injury; in the employment therefore of such remedies as bleeding and mercury, the intelligent practitioner will, after a little time, be better guided by his own experience of the case than by any general directions. It is a good rule in the treatment of all diseases, whether acute or chronic, when the remedies are affording little benefit, when the constitution rather than the disease seems to be yielding under them, to desist from them; this is a rule of common sense above the rules of art, and to which the latter ought to be subservient. I know no man whose knowledge of his profession is so exact, and whose opinion of a case is so infallible, that he may dispense with this rule.

"Lastly, there is a stage or a class of these cases which is chiefly benefited by restorative means, especially chalybeate waters. When the disease has lasted long, is not relieved by the above treatment, is accomanied by broken health, cold extremities, and a pale complexion, I have often known the disease cured by the waters of Tunbridge Wells or Bath; the patient gradually losing her pain, regaining the power of sitting up and going about, and acquiring a more healthy appearance. The plan, however, requires great caution, for where the jar of a carriage brings on pain, the journey will often do great mischief; and the chalybeate waters, which in some cases are so efficacious, are in others clearly pernicious. The probable effect of a journey may be known by a few drives, and the effect of the waters ought to be carefully watched; their influence on the pain and on the pulse is the best guide." 326.

Such is our author's description of this often intractable disease, and of the remedies which he has employed in the treatment. In his hands the result has been, that some who had been previously ill for several years, recovered after a few months and continued well, by strictly avoiding the exciting causes. Others, after a far longer treatment, experienced the same recovery; and this recovery was rendered permanent by the same long-continued caution. Others, again, after sooner or later recovering, have laid aside caution, indulged in unrestrained exercise and exertion, and have experienced a relapse as tedious and severe as the original attack. Lastly, his best efforts, in some cases, have alleviated, but not removed the disease, and he has had the mortification to see them, at the end of several years, little better than at the beginning.

"The older I grow, however, the fewer instances do I see of this hopeless condition of the disease, and some of which I had begun to despair, have slowly but ultimately recovered. I think it an important fact, that in the cases which remained uncured after many years, the patients had, for the relief of their pain, gradually accustomed themselves to a daily enormous allowance of opium." 327.

The paper terminates with a detail of three cases of the disease. But as we have given so full an analysis of this dissertation, we need not advert to the cases. The thanks of the profession are unquestionably due to Dr. Gooch, for this essay on a subject previously undescribed, and generally misunderstood, if not mistreated.

